# Initial multi-centre clinical experience with prone transpsoas lateral interbody fusion: Feasibility, perioperative outcomes, and lessons learned

**DOI:** 10.1016/j.xnsj.2021.100056

**Published:** 2021-03-04

**Authors:** Tyler G. Smith, Samuel A. Joseph, Benjamin Ditty, Rodrigo Amaral, Antoine Tohmeh, William R. Taylor, Luiz Pimenta

**Affiliations:** a Sierra Spine Institute, Roseville, CA, United States; b Joseph Spine Institute, Tampa, FL, United States; c The Spine Center at Joint Implant Surgeons of Florida, Naples, FL, United States; d Instituto de Patologia da Coluna, São Paulo, Brazil; e MultiCare Neurosurgery and Spine, Spokane, WA, United States; f University of California San Diego, San Diego, CA, United States

**Keywords:** LIF, LLIF, XLIF, PTP, Surgical position, Decubitus, Minimally invasive, MIS, Technique, Approach

## Abstract

**Background:**

Lateral interbody fusion (LIF) is traditionally performed with the patient in lateral decubitus, requiring repositioning to prone for adjunctive posterior procedures, or modifying traditional posterior techniques to be done while positioned lateral. The benefits of lateral anterior column access may be achievable with the patient prone, allowing for concomitant posterior techniques in a more familiar single-position setting.

**Methods:**

Prone transpsoas (PTP) access was outlined and vetted by a group of LIF-experienced spine surgeons. Early clinical experience included prospectively capturing procedural details and perioperative outcomes across a multi-centre cohort of clinicians to assess feasibility and to identify efficiencies and/or challenges.

**Results:**

Perioperative data was prospectively collected from 120 consecutive cases (176 levels) from 22 surgeons. Lateral exposure was achieved in an average 18 min/level, guided by triggered EMG; and retraction time averaged 25 min/level, with continued plexus monitoring via saphenous SSEP. Fixation was via percutaneous pedicle screws (65%), open pedicle screws (24%), other (11%). No re-positioning was required. Concomitant procedures facilitated by prone position included direct decompression (37%), treatment at L5-S1 (18%), posterior instrumentation revision (7%), and osteotomy/bony releases (9%). PTP procedure time, blood loss, and length of stay were consistent with established LIF experience. Challenges included patient movement with lateral instrument forces, retractor sag, stability of access relative to the patient, and surgeon ergonomics of the working channel. These challenges were overcome later in the experience through development of a specialized positioner and retractor system specific to this approach and a prescribed workflow developed by consensus of the surgeons.

**Conclusion:**

Initial multi-centre clinical experience suggests that PTP is not only feasible but creates efficiencies by allowing for single-position surgery maximizing both anterior and posterior column access and corrective techniques, with perioperative outcomes consistent with lateral decubitus experience. Learnings included the need for development of procedure-specific technologies and technique refinement.

## Introduction

Lateral interbody fusion (LIF) is a well published procedure which boasts important clinical advantages including the opportunity to effect an inherently biomechanically stable fusion environment [1], [2], [3], [4], with excellent disc and foraminal height restoration [5,6], and quick recovery owed to reduced morbidity of the minimally invasive (MIS) retroperitoneal access [7,8]. LIF as traditionally described is performed with the patient in the lateral decubitus position, coronally bent over a breaking table to gain access to the lumbar spine at levels L4–5 and above [9]. However, most lateral procedures include supplemental internal fixation via posterior pedicle screws, direct neural decompression, and/or other posterior releases to facilitate alignment correction. Those posterior procedures require, then, prone re-positioning – increasing time and risk of surgery, or the modification of traditional posterior techniques to be done while the patient remains positioned in lateral decubitus – introducing new technical challenges and risks [10,11].

A technique for approaching the lumbar spine laterally with the patient in the prone position was developed and has been recently described [12], theorizing that the benefits of lateral anterior column access may be achievable with the patient prone. Such single-position surgery may enable further benefits including intraoperative time savings without repositioning, simultaneous access to the lateral and posterior approaches, and utilization of widely available surgical suite equipment. The current case series is a prospective compilation of the initial multicenter case experience with prone transpsoas (PTP), captured to assess its feasibility, quantify its potential operative benefits and challenges through perioperative outcomes, and share the collective early learnings.

## Methods

In late 2018, a collective of spine surgeons experienced and interested in lateral approach surgery collaborated to identify challenges with the LIF technique and possible reasons for its limited adoption despite validated advantages. The study group identified the following limitations: (1) unfamiliar and time-consuming initial patient positioning; (2) the need for posterior procedures such as fixation, direct decompression, and/or releases in many patients, and so the requirement to reposition the patient to prone, increasing operative and anaesthesia time and risks; and (3) limitations to sagittal alignment correction while in the lateral position. Discussion led the group to question why lateral access to the lumbar spine required lateral positioning and to hypothesize that it could be achievable with the patient prone, which would address all identified concerns. Cadaveric experience followed, testing and iterating all aspects of the surgery from positioning and stability of the patient to retroperitoneal access, exposure requirements, and workflow.

Clinical experience expanded from there, leading to more than 600 PTP procedures performed by more than 50 spine surgeons between January 2019 and July 2020. That experience informs these claims in addition to the structured case data collected.

### Patient cohort

As clinical experience began, the collaborative sought to verify the feasibility and opportunities hypothesized and to support continued development and education by prospectively capturing procedural details and perioperative outcomes across the multi-centre cohort of clinicians. Non-identifying procedural and perioperative outcomes were documented on a procedure-specific case report form and included patient habitus, levels of surgery, side and position of PTP approach, depth of exposure, intraoperative monitoring, interbody devices used, type and timing of posterior fixation, other concomitant procedures performed, procedural and total operative time and blood loss, notation of any intraoperative challenges, and length of hospital stay.

Data from multi-centre case report forms were consolidated and analysed using summary statistics. Qualitative experience was shared throughout and led to continued iteration of technique and technologies to address challenges and optimize efficiencies.

### Surgical technique

The surgical technique, illustrated by Pimenta et al. [12], is consistent with prior LIF descriptions, apart from prone positioning on a Jackson frame-type bed using a procedure-specific positioner and retractor that optimize exposure and efficiency while the patient is in the prone position. The technique as described in Pimenta et al. continued to evolve with early clinical experience toward the iterative development of improved procedural systems and steps, as it became clear that simply using the systems historically developed for lateral decubitus LIF would not suffice. Such has become the differentiation between the PTP procedure as described herein and other reports of “prone lateral.”

**Fig. 1:**
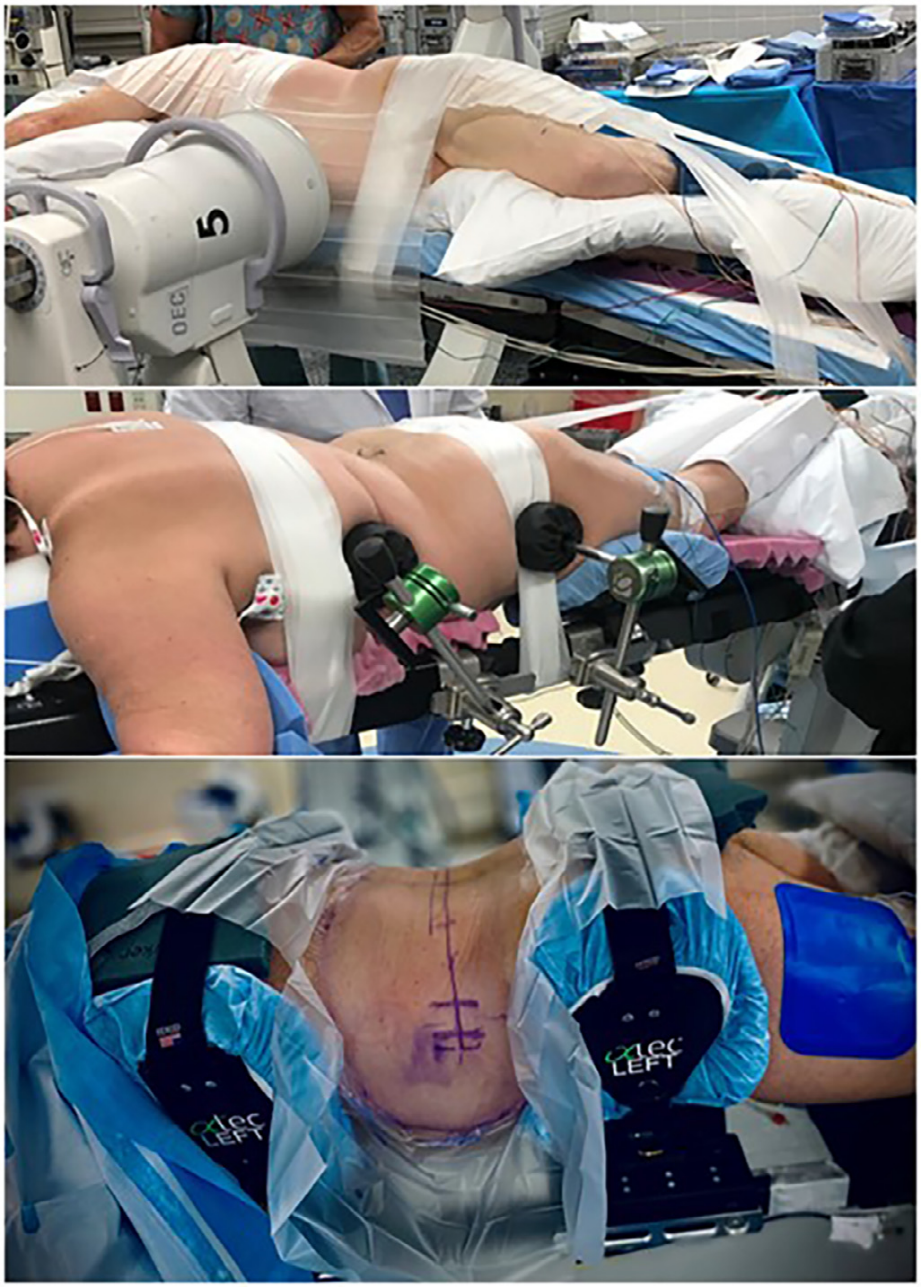
Patient positioning for lateral surgery. Top: traditional lateral decubitus, taped to a breaking surgical table; Middle: prone – early experience, taped to Jackson frame-style surgical table and using contralateral supports to counter lateral instrument forces; Bottom: PTP as evolved, positioned using customized system to more robustly stabilize patient and control the torso while eliminating the use of tape.

Positioning, for example, had to address patient stability under new lateral forces, while also allowing for abdominal content excursion, pannus management, optimized sagittal curvature, and coronal bending when necessary, as well as consideration of hip extension and the anatomical consequences of the position and tension on the psoas muscle and lumbar plexus (Fig. 1).

**Fig. 2:**
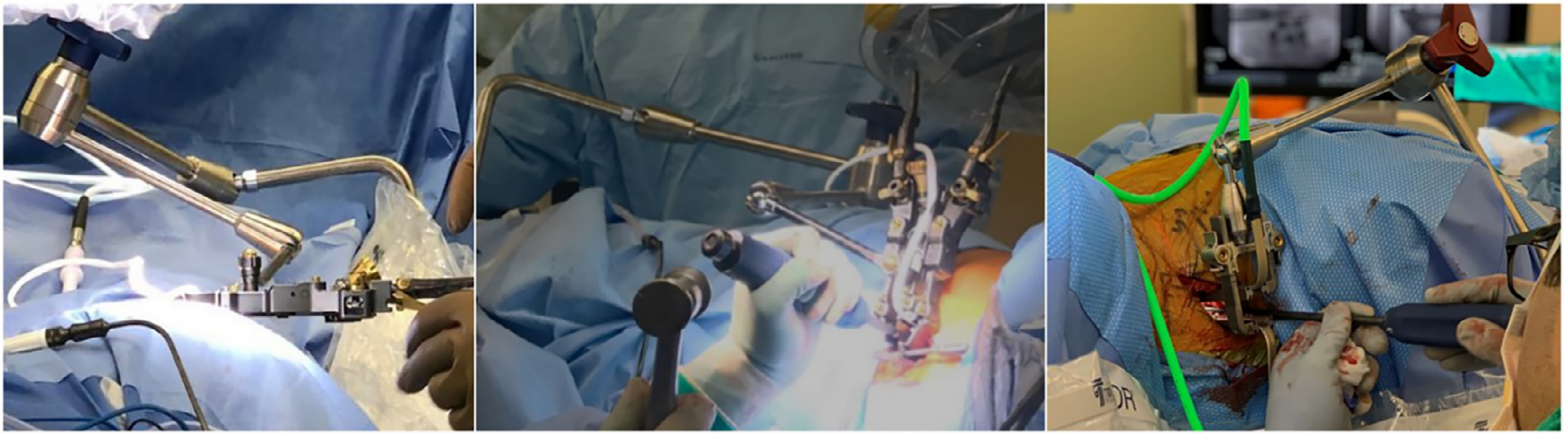
Retractor orientations for lateral surgery. Left: traditional lateral decubitus, showing typical LIF retractor orientated to visualize down from top, its stability assisted by gravity; Middle: prone – early experience, showing same LIF retractor orientated to visualize from side, its stability challenged by gravity; Right: PTP as evolved, showing customized retractor optimized for prone orientation through reduced weight, simplified and more rigid construction and articulating arm attachment.

Retroperitoneal and transpsoas access also had to be reconsidered to account for gravitational effect and visual and tactile function and ergonomics of the approach. Experience led to retractor redesign as a two-bladed anterior-posterior exposure, while minimizing weight and maximizing rigidity to avoid anterior migration (Fig. 2).

**Fig. 3:**
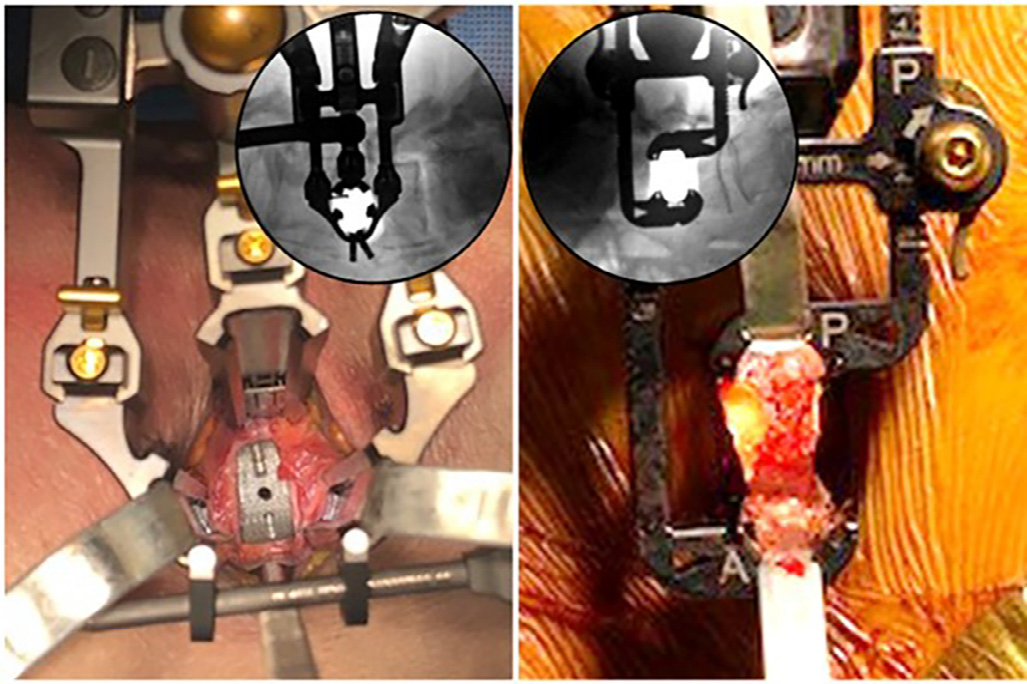
Exposure for lateral surgery (bottom of images is anterior, top is posterior). Exposure need only be as wide as endplate-to-endplate in the cranio-caudal dimension and providing as much access to the lateral aspect of the disc space as is safely feasible in the anterior-to-posterior dimension. That posterior margin is typically determined by localization of the plexus during the approach with tEMG, which, can push the starting point more anterior, limiting posterior exposure. Left: typical LIF three-bladed retractor exposure. Right: PTP exposure using customized two-bladed retractor with independent expansion in the anterior and posterior directions.

The two-bladed retractor design facilitates exposure through independent anterior and posterior movement, enabling a more forgiving starting position closer to mid-disc than traditional posterior targeting, where the location of the plexus can be a challenge at the lowest levels (Fig. 3).

**Fig. 4:**
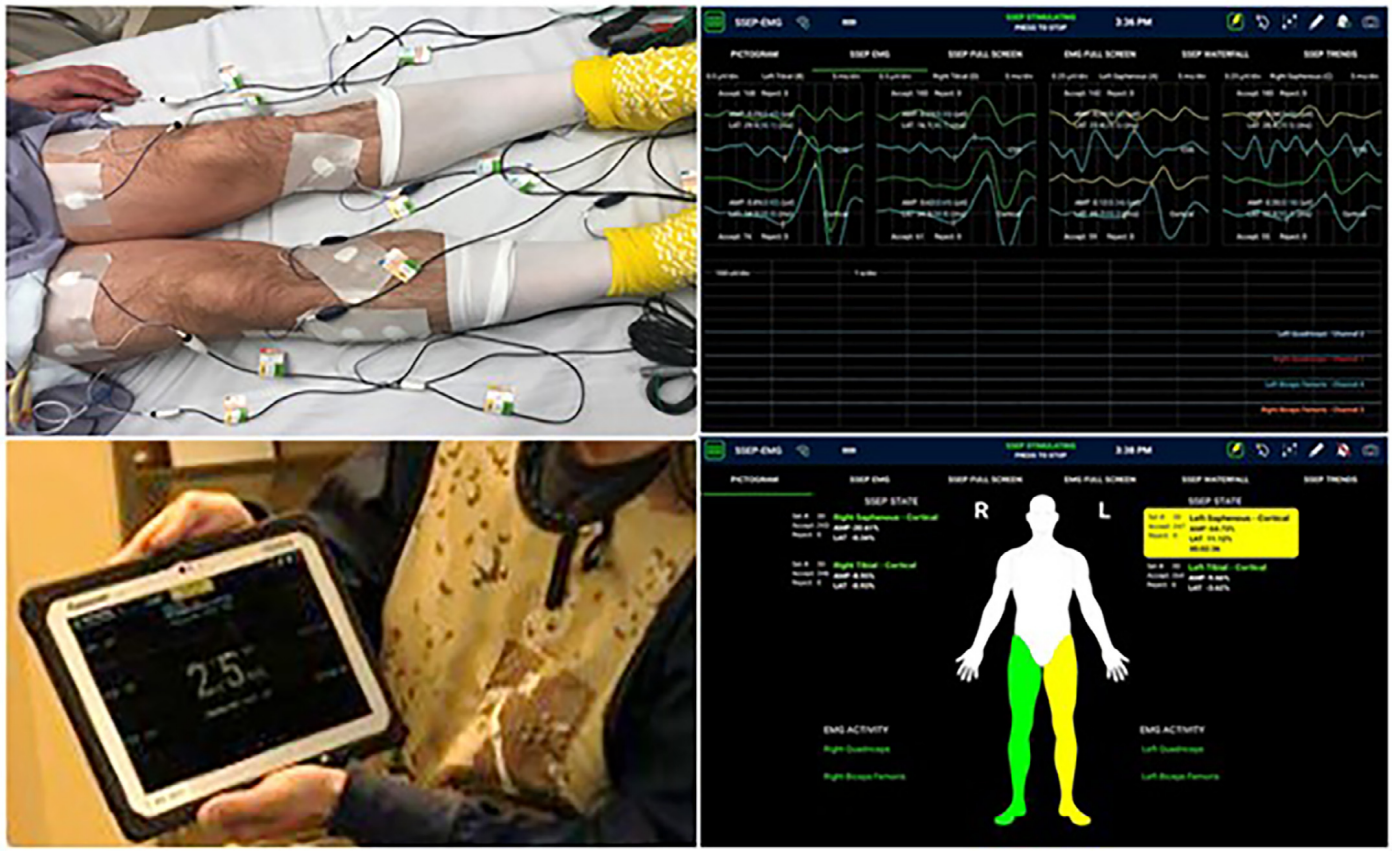
Intraoperative monitoring of nerve location via triggered EMG (tEMG) and saphenous SSEP.

Lumbar plexus safety remained a concern. However, prone positioning with the hips neutral to extended seems to lengthen the psoas muscle and draw it and the plexus with it more posteriorly [13,14]. This, combined with a mid-disc initial docking and customized A-P exposure from there, have increased the authors’ comfort with access at L4–5. In addition, the PTP procedure has benefited from the advancement of saphenous SSEP to monitor femoral nerve health through the entirety of the procedure (in addition to tEMG for nerve identification during the approach) (SafeOp™, Alphatec Spine) (Fig. 4).

## Results

Perioperative data was prospectively collected from 120 consecutive cases from 22 surgeons, marking the early experience and learning curve of each, spanning the time period from January 2019 through July 2020. All procedures, regardless of patient size (mean BMI 33; range 21–46) were feasible; i.e., none were aborted due to size limitations. PTP was performed at a total of 176 levels. The L4–5 disc space was included in 68% of patients, and procedures spanned as cranially as L1–2.

Transpsoas positioning of the retractor was most commonly between the posterior 4th and 3rd quadrants of the disc, guided by triggered EMG, and with the addition of continued plexus monitoring via saphenous SSEP. Stable exposure was achieved in an average 18 min from initial skin incision; retraction time averaged 25 min per level. Interbody devices used were primarily porous titanium (66%; IdentiTi^Ⓡ^, Alphatec Spine), followed by PEEK (34%; Transcend™, Alphatec Spine).

Fixation type was most commonly via percutaneous pedicle screws (65%), followed by open pedicle screws (24%), spanning up to 9 levels from as cranial as T8 to the pelvis caudally. No re-positioning was required in any case. Fixation was most commonly performed sequentially after PTP interbody work, but was completed prior to PTP in some cases where posterior revision, releases, and/or reduction was thought to facilitate the anterior work. In some cases where multiple surgeons were working simultaneously, posterior work was performed concurrent with the PTP. Other concomitant posterior procedures facilitated by prone position included direct decompression (37%), TLIF at L5-S1 (18%), revision of posterior instrumentation (7%), and osteotomy/bony releases (9%).

PTP procedure time averaged 34 min per level out of a total average operative time of 3 h 14 min. Blood loss averaged 85 cc for PTP and 240 cc overall. Length of stay averaged 2.2 days (range 1–5 days). Intraoperative complications included inadvertent ALL release in 2 (1.7%) with no consequence, and localized segmental bleeding – controlled intraoperatively – in 5 (4.2%). Postoperative complaints included reports of mild transient thigh symptoms in 8 (6.7%).

**Fig. 5:**
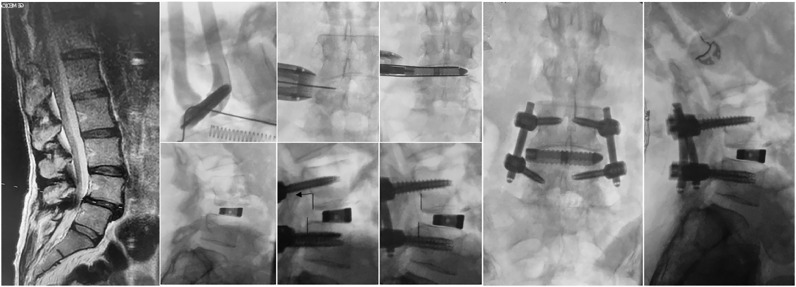
Case example of L4–5 PTP LIF with MIS posterior fixation all performed in single position (prone).

A representative case example is shown in Fig. 5. A 52-year-old female presented with low back and bilateral leg pain, consistent with preoperative imaging showing unstable L4–5 spondylolisthesis with lateral recess and sub-articular stenosis. L4–5 PTP with MIS posterior fixation was performed. Due to the slip, and associated anterior position of the nerves as identified by tEMG, a more anterior starting position was required; however, full lateral exposure was achieved by preferential expansion of the posterior blade. Interbody correction with porous titanium cage (IdentiTi, ATEC Spine) was followed by further reduction through the posterior construct (Invictus SingleStep, ATEC Spine).

## Discussion

### Learnings

One of the first challenges identified in the authors’ experience with prone lateral using traditional systems was patient stability. Counterintuitively, prone positioning used for the majority of modern spine surgeries is thought of as inherently stable. However, when approaching from the side, the generation of lateral instrument forces during interbody preparation and implant placement can cause movement of the patient on the table and/or of the spine within the torso, causing the table-mounted retractor to effectively pull away from the surface of the spine and risk soft tissue encroachment into the exposure. Retractor stabilizers such as screw shim anchors were considered and tried, but ultimately were not required as the design of a robust procedure-specific patient positioner obviated the issue (Fig. 1).

That positioner also had to allow for coronal bend when necessary to improve access around the iliac crest at L4–5 or the rib at L1–2. A study of asymptomatic volunteers imaged in both lateral decubitus position on a breaking Amsco table and subsequently prone on a Jackson frame showed that the positioner improved access to L4–5 compared to lateral decubitus, and also improved lordosis [15].

The positioner also had to accommodate varying body habitus representative of the generalized patient population and manage excess abdominal tissue. This is achieved with nylon straps rather than tape on the skin, which also streamlines set-up. Due to the flattening of abdominal girth in prone, the depth of the exposure can be greater than might otherwise be in lateral decubitus where the pannus falling anteriorly stretches the lateral skin taut. The coronal bend feature of the positioner helps this as well, but careful finger dissection of the retroperitoneal space can be a challenge, and longer retractor blades are sometimes required.

Longer retractor blades have traditionally been more challenging because of their propensity to flex at the deep margins of the exposure creating a diminishing cone. PTP retractor development took this into consideration as well as the overall weight and positional rigidity to avoid anterior migration while providing an optimized exposure over the lateral aspect of the disc (Fig. 2, Fig. 3). This required redesign of the geometry of the table-mounted retractor arm as well as integration of the mounting point onto the bolster itself instead of the table.

The ergonomics of visual and tactile access to the disc were also considered and evaluated. The operating surgeon may sit or stand as preferred, but in either position, it is the authors’ recommendation that the bed be tilted away for line-of-sight access to the disc space. This manoeuvre should be performed, however, only after achieving orthogonality during retractor positioning, in order to maintain a safe and biomechanically optimal access to the disc space. Orthogonality with the disc space is maintained throughout disc work by ensuring instruments pass parallel with the retractor blades.

Other efficiencies that were notably gained during the collective experience include the ability to perform any and all necessary posterior work (before, after, or simultaneous with lateral), saving flip time and expanding clinical decision-making options, such as in which patients direct or indirect decompression is warranted. The cohort captured included short and long posterior constructs (some navigated), decompressions, osteotomies, revisions, and interbody procedures at L5-S1 from a posterior approach. Prone positioning also enabled both right- and left-sided lateral approaches to optimize correction of a double coronal curve. Sagittal corrections were also optimized, as it has been the authors’ experience that prone positioning provides a significant improvement in positional lordosis, facilitating disc preparation and the use of 15 and even 20° lordotic cages without ALL release [16,17].

In this way, the PTP procedure may expand the utility of the LIF procedure – with its advantage for MIS anterior column correction – to a broader array of surgical applications, including complex deformity, where LIF has been slowly adopted.

## Conclusion

Initial multicenter clinical experience suggests that PTP is not only feasible but creates efficiencies by allowing for single-position surgery maximizing both anterior and posterior column access and corrective techniques, with perioperative outcomes consistent with lateral decubitus experience. Learnings included need for development of procedure-specific technologies as well as refinement of technique details.
